# Supplementary material for: A diminutive new basilosaurid whale reveals the trajectory of the cetacean life histories during the Eocene
Source: Commun Biol. 2023 Aug 10;6:707. doi: 10.1038/s42003-023-04986-w (PMC10415296; doi:10.1038/s42003-023-04986-w)
Supplement: Supplementary file 6 — Supplementary Data 3 [file 42003_2023_4986_MOESM6_ESM.zip › Supplementary Data 3/Supplementary Data 2_BTD_ASR/trait_0030_tree.plot.pdf]

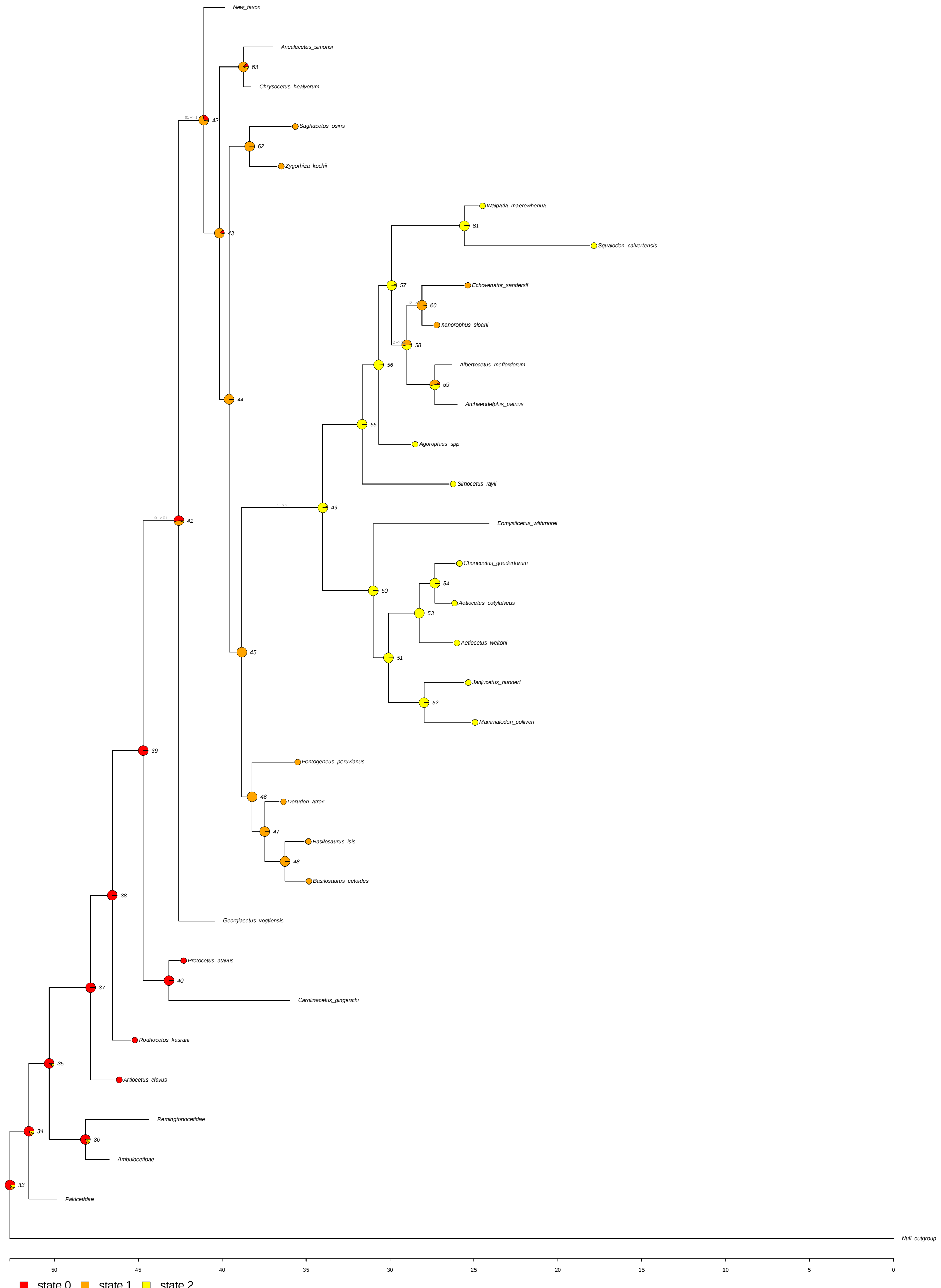

New_taxon
Ancalecetus_simonsi
63
Chrysocetus_healyorum
42
Saghacetus_osiris
62
Zygorhiza_kochii
Waipatia_maerewhenua
61
Squalodon_calvertensis
43
57
Echovenator_sandersii
60
Xenorophus_sloani
58
56
Albertocetus_meffordorum
59
Archaeodelphis_patrius
44
55
Agorophius_spp
Simocetus_rayii
49
1 --> 2
0 --> 01
41
Eomysticetus_withmorei
Chonecetus_goedertorum
54
50
Aetiocetus_cotylalveus
53
Aetiocetus_weltoni
45
51
Janjucetus_hunderi
52
Mammalodon_colliveri
39
Pontogeneus_peruvianus
46
Dorudon_atrox
47
Basilosaurus_isis
48
Basilosaurus_cetoides
38
Georgiacetus_vogtlensis
Protocetus_atavus
40
37
Carolinacetus_gingerichi
Rodhocetus_kasrani
35
Artiocetus_clavus
Remingtonocetidae
34
36
Ambulocetidae
33
Pakicetidae
Null_outgroup
50
45
40
35
30
25
20
15
10
5
0
state 0
state 1
state 2
